# Supplementary material for: Memory and relatedness of transcriptional activity in mammalian cell lineages
Source: Nat Commun. 2019 Mar 14;10:1208. doi: 10.1038/s41467-019-09189-8 (PMC6418128; doi:10.1038/s41467-019-09189-8)
Supplement: Supplementary file 1 — Supplementary Information [file 41467_2019_9189_MOESM1_ESM.pdf]

Memory and relatedness of transcriptional activity in mammalian cell lineages - Supplementary Information

Phillips et al.

1 Supplementary Note 1

1.1 A mathematical model of transcriptional fluctuations in populations of dividing cells

In this section we describe the mathematical model of transcriptional fluctuations in dividing cells. As outlined in the main text, the model has two variables per single cell that together describe stochastic reporter dynamics: the transcriptional activity S that acts as a source for the transcriptional reporter R. We also show how cell division is accounted for in the initial conditions of the model. The two variable model uses parameters that are specific to each cell, and we subsequently describe how a hierarchical model allows estimation of population level parameters describing the distribution of cell-specific parameters. The parings of cells used are either sister cells, mother-daughter cells or randomised. Following the model definition we describe how Gaussian processes and Hamiltonian Markov Chain Monte Carlo (HMC) are used to infer the parameters of the model from the experimental bioluminescent reporter time series.

1.1.1 Model definition

For a pair of cells (labelled $i \in \{1, 2\}$), each cell has two variables: a variable R representing the total levels of measured bioluminescent reporter and a variable S that represent transcriptional activity and controls the production of R. The dynamics of the transcriptional activity and reporter for cell i is represented with the following system of stochastic differential equations:

$$\frac{dS_i(t)}{dt} = -\frac{1}{\tau_S}(S_i(t) - \mu_i) + \epsilon_i(t), \quad i \in \{1, 2\} \tag{1}$$

$$\frac{dR_i(t)}{dt} = \frac{\ln(2)}{\tau_R}(S_i(t) - R_i(t)) + \eta_i(t). \quad i \in \{1, 2\} \tag{2}$$

where μ_i is the cell-specific mean level of S_i and τ_S sets the timescale of fluctuations around μ_i (*i.e.* slow or rapid fluctuations for large or small τ_S, respectively). The distribution of the cell-specific means μ_i is further modelled at the population level (see Section 1.1.4). Note that to save parameters, mRNA is not explicitly modelled; we estimated the net reporter half-life (which thus depends on both the mRNA and protein half-life) by blocking transcription with actinomycin D and by fitting a first order exponential decay to the decrease in reporter levels (values shown in in Supp Fig. 5). The terms ϵ_i and η_i are taken as Gaussian white noise terms with zero mean and covariances

$$\langle \epsilon_i(t)\epsilon_i(t') \rangle = \frac{2(\sigma_{S,i})^2}{\tau_S}\delta(t - t'), \quad i \in \{1, 2\} \tag{3}$$

$$\langle \epsilon_i(t)\epsilon_j(t') \rangle = \rho_{\text{SIS}}\frac{2(\sigma_{S,i}\sigma_{S,j})}{\tau_S}\delta(t - t'), \quad i \neq j \text{ (sisters)} \tag{4}$$

$$\langle \eta_i(t)\eta_i(t') \rangle = \frac{2\ln(2)\sigma_R^2}{\tau_R}\delta(t - t'), \quad i \in \{1, 2\} \tag{5}$$

$$\langle \epsilon_i(t)\eta_i(t') \rangle = \langle \epsilon_i(t)\eta_j(t') \rangle = \langle \eta_i(t)\eta_j(t') \rangle = 0, \quad i \neq j \text{ (sisters)} \tag{6}$$

where $\sigma_{S,i}$ and $\sigma_{R,i}$ are cell-specific parameters that control the noise amplitude on the dynamics of S_i and R_i, respectively. These covariances were normalised by the timescales τ_S and τ_R to simplify the covariances of S_i and R_i in the stationary state and reduce parameter correlation during inference. The parameter $\rho_{\text{SIS}} \in [-1, 1]$ represents the correlation in transcriptional noise between sister ells, and including the correlation in transcriptional noise (ρ_{SIS}) between sister cells is a phenomenological means to produce two sister cells with correlated trajectories, where the similarity can be tuned with ρ_{SIS}. When pairs of mother-daughter cells are analysed, we use the notation ρ_{MD} instead. The reporter level noise term $\eta_i(t)$ represents effective noise combining both molecular fluctuations in reporter levels as well as experimental noise and is assumed to be independent between two cells.

1.1.2 Model solution

Our model consists of a system of two linear stochastic differential equations for each cell, and if the initial conditions of the two variables are normally distributed then the model is a Gaussian process. Samples from a Gaussian process taken at discrete times have a multivariate normal distribution, and hence the model is fully specified by the mean at each time point and the covariance between all time points. Considering two cells, the model can be written equivalently in matrix form, where the variables form the column vector $\mathbf{x} = [S_1, S_2, R_1, R_2]^T$.

$$\frac{d}{dt}\mathbf{x}(t) = -A(\mathbf{x}(t) - \boldsymbol{\phi}) + \boldsymbol{\zeta}(t), \tag{7}$$

where

$$\boldsymbol{\phi} = \begin{bmatrix} \mu_1 \\ \mu_2 \\ 0 \\ 0 \end{bmatrix}, \tag{8}$$

$$-A = \begin{bmatrix} -\frac{1}{\tau_S} & 0 & 0 & 0 \\ 0 & -\frac{1}{\tau_S} & 0 & 0 \\ \frac{\ln(2)}{\tau_R} & 0 & -\frac{\ln(2)}{\tau_R} & 0 \\ 0 & \frac{\ln(2)}{\tau_R} & 0 & -\frac{\ln(2)}{\tau_R} \end{bmatrix}, \tag{9}$$

$$\langle \boldsymbol{\zeta}(t)\boldsymbol{\zeta}^T(t') \rangle = \begin{bmatrix} \frac{2(\sigma_{S,i})^2}{\tau_S} & \rho_{\text{SIS}}\frac{2\sigma_{S,i}\sigma_{S,j}}{\tau_S} & 0 & 0 \\ \rho_{\text{SIS}}\frac{2\sigma_{S,i}\sigma_{S,j}}{\tau_S} & \frac{2(\sigma_{S,j})^2}{\tau_S} & 0 & 0 \\ 0 & 0 & \frac{2\ln(2)(\sigma_{R,i})^2}{\tau_R} & 0 \\ 0 & 0 & 0 & \frac{2\ln(2)(\sigma_{R,j})^2}{\tau_R} \end{bmatrix} \delta(t - t'). \tag{10}$$

The solution is (adapted from [1])

$$\mathbf{x}(t) = e^{-At}\mathbf{x}(0) + \int_0^t e^{-A(t-t')}A\boldsymbol{\phi}dt' + \int_0^t e^{-A(t-t')}\boldsymbol{\zeta}(t)dt', \tag{11}$$

and the mean and covariance functions are given by

$$\langle \mathbf{x}(t) \rangle = e^{-At}\langle \mathbf{x}(0)\rangle + \int_0^t e^{-A(t-t')}A\boldsymbol{\phi}dt', \tag{12}$$

$$\langle \mathbf{x}(t_1)\mathbf{x}^T(t_2) \rangle = e^{-At_1}\langle \mathbf{x}(0)\mathbf{x}^T(0)\rangle e^{-A^Tt_2} + \langle \int_0^{\min(t_1,t_2)} e^{-A(t_1-t')}\boldsymbol{\zeta}(t)\boldsymbol{\zeta}^T(t')e^{-A^T(t_2-t')}dt' \rangle. \tag{13}$$

where $\langle \mathbf{x}(0)\rangle$ is the vector representing the initial mean levels of S and R at time $t = 0$ while $\langle \mathbf{x}(0)\mathbf{x}^T(0)\rangle$ is the matrix of initial covariances at time $t = 0$ (defined below). Once initial conditions have been specified, the covariance function (Equation 13) was found in function of the model parameters using matrix exponentiation within the symbolic toolbox of MATLAB. For two cells the model is then fully specified by the mean functions $\langle R_1(t)\rangle$ and $\langle R_2(t)\rangle$, the covariance functions $\langle R_1(t_1)R_1(t_2)\rangle$ and $\langle R_2(t_1)R_2(t_2)\rangle$ and the cross-covariance function $\langle R_1(t_1)R_2(t_2)\rangle$.

1.1.3 Initial conditions

The mean ($\langle \mathbf{x}(0)\rangle$) and covariance ($\langle \mathbf{x}(0)\mathbf{x}^T(0)\rangle$) of the model needs to be specified for all variables immediately following cell division (*i.e.* at time $t = 0$). To define the initial state of each cell following division we calculate the steady state distribution of S and R and then assume that R is divided by two, reflecting the fact that we measure the total levels of transcriptional reporter, which are approximately halved at cell division. Under this assumption the initial means of variables S and R for cell $i \in \{1, 2\}$ are given by

$$\langle S_i(0)\rangle = \mu_i, \tag{14}$$

$$\langle R_i(0)\rangle = \frac{\mu_i}{2}, \tag{15}$$

and the initial covariances within each sister cell are then given by

$$\langle S_i(0)S_i(0)\rangle = \sigma^2_{S,i}, \quad i \in \{1, 2\} \tag{16}$$

$$\langle R_i(0)R_i(0)\rangle = \left(\frac{\sigma^2_{S,i}\ln(2)}{\tau_R} + \frac{\sigma^2_{R,i}\ln(2)}{\tau_R} + \frac{\sigma^2_{R,i}}{\tau_S}\right)/4\left(\frac{\ln(2)}{\tau_R} + \frac{1}{\tau_S}\right), \quad i \in \{1, 2\} \tag{17}$$

$$\langle S_i(0)R_i(0)\rangle = \left(\frac{\sigma^2_{S,i}\ln(2)}{\tau_R}\right)/2\left(\frac{\ln(2)}{\tau_R} + \frac{1}{\tau_S}\right). \quad i \in \{1, 2\} \tag{18}$$

We assume that the initial covariances between sister cells $\langle S_1(0)S_2(0)\rangle$, $\langle R_1(0)R_2(0)\rangle$ and $\langle S_1(0)R_2(0)\rangle$ are zero during the inference procedure. Note that the variables R_1 and R_2 can still be correlated due to correlation in the cell-specific means μ_1 and μ_2, described in Section 1.1.4. Later, when the objective is to predict the decay in correlation over the cell cycle, the initial correlation between sister cells is chosen to match the empirical initial correlation from the data at time $t = 0$ (see Section 1.2.8).

1.1.4 Population model of cell-specific parameters

We use a hierarchical modelling approach whereby the cell-specific parameters μ_i, $\sigma_{S,i}$ and $\sigma_{R,i}$ are obtained from a higher-level population distribution. We assume that the cell-specific mean (μ_i) and variance parameters ($\sigma_{S,i}$ and $\sigma_{R,i}$) are log-normally distributed across the population. These three log-normal distributions

for μ_i, $\sigma_{S,i}$ and $\sigma_{R,i}$ each possess a mean parameter that we denote m, Λ_S, Λ_R, respectively, and which set the global average in log space. The variance of the log-normal distribution is parameterised with s^2, Σ_S, Σ_R, and acts to control the variability across cells. We also introduce parameter λ_{SIS} to quantify the correlation in mean transcriptional activities between pairs of sister cells (i.e. the correlation between μ_1 and μ_2 for pairs of sisters across the population). We use the notation λ_{MD} when the correlation refers to mother-daughter pairs. Together the population distributions of μ_i, $\sigma_{S,i}$ and $\sigma_{R,i}$ are represented as follows:

$$\log\left(\begin{bmatrix}\mu_i\\ \mu_j\end{bmatrix}\right) \sim \mathcal{N}\left(\begin{bmatrix}m\\ m\end{bmatrix}, \begin{bmatrix}s^2 & \lambda_{\text{SIS}}s^2\\ \lambda_{\text{SIS}}s^2 & s^2\end{bmatrix}\right), \tag{19}$$

$$\log\left(\begin{bmatrix}\sigma_{S,i}\\ \sigma_{S,j}\end{bmatrix}\right) \sim \mathcal{N}\left(\begin{bmatrix}\Lambda_S\\ \Lambda_S\end{bmatrix}, \begin{bmatrix}\Sigma_S & 0\\ 0 & \Sigma_S\end{bmatrix}\right), \tag{20}$$

$$\log\left(\begin{bmatrix}\sigma_{R,i}\\ \sigma_{R,j}\end{bmatrix}\right) \sim \mathcal{N}\left(\begin{bmatrix}\Lambda_R\\ \Lambda_R\end{bmatrix}, \begin{bmatrix}\Sigma_R & 0\\ 0 & \Sigma_R\end{bmatrix}\right). \tag{21}$$

1.2 Inferring the model parameters using transcriptional reporter dynamics in dividing cells

The aim is to estimate the parameters of the model for each gene individually, given the global constraints. For a given set of parameters there are three terms that need to be evaluated within our hierarchical inference scheme: 1) the likelihood of the fluctuating time series 2) the population level 3) the prior/hyperprior level. For sampling the parameters we use HMC, which also requires the gradient of each term with respect to each parameter. We now describe how each term is calculated before showing how they are combined for parameter sampling.

1.2.1 Likelihood calculation using single-cell time series

At the lowest level of our hierarchical model we compute the likelihood that pairs of single-cell bioluminescent time series were generated by our gene expression model for a given set of parameters (the likelihood function).

For each pair of cells there is a cell 1 and cell 2. The levels of reporter are measured every 5 minutes, but the overall length of the measurement can be different between two cells due to differences in cell-cycle length. If the total number of data points in cell 1 is N_1 then the time series of cell 1 is $\mathbf{y}_1 = [y_1(t_1), y_1(t_2), ..., y_1(t_{N_1})]^T$ and similarly if the total number of data points in cell 2 is N_2 then the time series of cell 2 is $\mathbf{y}_2 = [y_2(t_1), y_2(t_2), ..., y_2(t_{N_2})]^T$. The measurements of both cells from pair p can be combined into a single vector as $\mathbf{y}_p = [y_1(t_1), ..., y_1(t_{N_1}), y_2(t_1), ..., y_2(t_{N_2})]^T$. The corresponding vector of time points can similarly be written as $\mathbf{t}_p = [t_1, ..., t_{N_1}, t_1, ..., t_{N_2}]^T$. Our task is then to calculate the likelihood of observing $\mathbf{y}_p$ given the model and parameters. The total set of parameters for each pair p consist of $\theta_p = \{\tau_S, \rho_{\text{SIS}}, \mu_1, \mu_2, \sigma_{S,1}, \sigma_{S,2}, \sigma_{R,1}, \sigma_{R,2}\}$, where τ_S and ρ_{SIS} are global parameters and the other

parameters are all cell-specific. As the model represents a Gaussian process, the observations for pair p are described as a joint multivariate normal distribution and the log-likelihood can therefore be written as [2]

$$\log p(\mathbf{y}_p|\mathbf{t}_p, \theta_p) = -\frac{1}{2}(\mathbf{y}_p - \mathbf{m}_p)^T K_p^{-1}(\mathbf{y}_p - \mathbf{m}_p) - \frac{1}{2}\log|K_p| - \frac{N_1 + N_2}{2}\log 2\pi, \tag{22}$$

where the vector $\mathbf{m}_p$ describes the expected values of the reporter levels at each time point from the model *i.e.* $\mathbf{m}_p = [\langle R_1(t_1)\rangle, ..., \langle R_1(t_{N_1})\rangle, \langle R_2(t_1)\rangle, ..., \langle R_2(t_{N_2})\rangle]^T$. The covariance matrix K_p defines the covariance between each element of the measured values in $\mathbf{y}_p$ and has the following block structure

$$K_p(t, t') = \begin{bmatrix} K(R_1, R_1) & K(R_1, R_2) \\ K(R_2, R_1) & K(R_2, R_2) \end{bmatrix}, \tag{23}$$

where each block is a covariance matrix with each element $K(R_i, R_j)_{k,l} = \langle R_i(t_k) R_j(t_l)\rangle$ (for $i, j \in \{1, 2\}$). As the length of $\mathbf{y}_1$ and $\mathbf{y}_2$ can differ, the size of each block $K(R_i, R_j)$ is $N_i \times N_j$.

To compute the log-likelihood for each pair of cells (Equation 22) we use Algorithm 2.1 from [2], which we now describe. Calculating the log-likelihood involves the inverse of the covariance matrix $K_p(t, t')$ (Equation 23). Covariance matrices are both symmetric and positive definite, and linear systems involving covariance matrices can be solved efficiently using the Cholesky decomposition. The Cholesky decomposition of the covariance matrix K decomposes K into a product of a lower triangular matrix L and its transpose L^T

$$K = LL^T, \tag{24}$$

where L is the Cholesky factor, which is a lower triangular matrix with real and positive diagonal entries. Simplifying the notation by denoting $\mathbf{y} = \mathbf{y}_p - \mathbf{m}_p$, the problem of finding $\mathbf{y}^T K^{-1}\mathbf{y}$ in Equation 22 can then be simplified by first solving $K\boldsymbol{\alpha} = \mathbf{y}$ for $\boldsymbol{\alpha}$. This can be achieved by first solving the lower triangular system $L\boldsymbol{\beta} = \mathbf{y}$ by forward substitution and then the upper triangular system $L^T\boldsymbol{\alpha} = \boldsymbol{\beta}$ by back substitution. The solution for $\boldsymbol{\alpha}$ is therefore given by $\boldsymbol{\alpha} = L^T\backslash(L\backslash\mathbf{y})$, where under the backslash notation $K\backslash\mathbf{y}$ is the vector $\boldsymbol{\alpha}$ that solves $K\boldsymbol{\alpha} = \mathbf{y}$. The asymptotic runtime of the Cholesky factorisation is $O(N^3)$ for N number of data points, whereas the forward and backward substitutions require $O(N^2)$ operations. Once the Cholesky factor L and $\boldsymbol{\alpha}$ have been obtained, the likelihood of each cell is then computed using

$$\begin{aligned} \log p(\mathbf{y}_p|\mathbf{t}_p, \theta_p) &= -\frac{1}{2}\mathbf{y}^T K_p^{-1}\mathbf{y} - \frac{1}{2}\log|K_p| - \frac{N_1 + N_2}{2}\log 2\pi \\ &= -\frac{1}{2}\mathbf{y}^T\boldsymbol{\alpha} - \sum_i \log(L_{ii}) - \frac{N_1 + N_2}{2}\log 2\pi \end{aligned} \tag{25}$$

The HMC scheme also requires gradients of the log-likelihood for each parameter. The partial derivative of the log-likelihood with respect to parameter θ_j for parameters affecting the mean of the Gaussian process is

$$\frac{\partial}{\partial\theta_j}\log p(\mathbf{y}_p|\mathbf{t}_p, \theta_p) = \frac{\partial\mathbf{m}_p^T}{\partial\theta_j} L^T\backslash(L\backslash\mathbf{y}), \tag{26}$$

and for parameters that affect the covariance matrix K_p the partial derivative of the likelihood with respect to parameter θ_j is

$$\frac{\partial}{\partial \theta_j} \log p(\mathbf{y}_p|\mathbf{t}_p,\theta_p) = -\frac{1}{2}\mathbf{y}^T K_p^{-1} \frac{\partial K_p}{\partial \theta_j} K_p^{-1}\mathbf{y} - \frac{1}{2}\,\mathrm{Tr}(K_p^{-1}\frac{\partial K_p}{\partial \theta_j}) \ . \tag{27}$$

The combined dataset for a total of n_{pairs} pairs of cells is $\mathcal{D} = \{\mathbf{y}_p\}_{p=1}^{n_{\text{pairs}}}$, and the full log-likelihood $\log(\mathcal{L}(\mathcal{D}|\theta))$ is then the product of the likelihood over all pairs of cells

$$\log(\mathcal{L}(\mathcal{D}|\theta)) = \sum_{i=1}^{n_{\text{pairs}}} \log p(\mathbf{y}_p|\mathbf{t}_p,\theta_p) \tag{28}$$

1.2.2 Population level

For each pair p of cells there are the cell-specific parameters $\boldsymbol{\nu}_p = [\mu_1,\mu_2,\sigma_{S,1},\sigma_{S,2},\sigma_{R,1},\sigma_{R,2}]^T$. The probability of observing these parameters are given by Equations (19-21) and are dictated by the population parameters $\Delta = \{m,\Lambda_S,\Lambda_R,s,\lambda_{\text{SIS}},\Sigma_S,\Sigma_R\}$. Combining Equations (19-21) together, the probability of observing the cell-specific parameters for each cell is

$$\log\left(\begin{bmatrix}\mu_1\\ \mu_2\\ \sigma_{S,1}\\ \sigma_{S,2}\\ \sigma_{R,1}\\ \sigma_{R,2}\end{bmatrix}\right) \sim \mathcal{N}\left(\begin{bmatrix}m\\ m\\ \Lambda_S\\ \Lambda_S\\ \Lambda_R\\ \Lambda_R\end{bmatrix}, \begin{bmatrix} s^2 & \lambda_{\text{SIS}}s^2 & 0 & 0 & 0 & 0\\ \lambda_{\text{SIS}}s^2 & s^2 & 0 & 0 & 0 & 0\\ 0 & 0 & \Sigma_S & 0 & 0 & 0\\ 0 & 0 & 0 & \Sigma_S & 0 & 0\\ 0 & 0 & 0 & 0 & \Sigma_R & 0\\ 0 & 0 & 0 & 0 & 0 & \Sigma_R\end{bmatrix}\right) \tag{29}$$

Writing the mean of this lognormal distribution (*i.e.* $[m,m,\Lambda_S,\Lambda_S,\Lambda_R,\Lambda_R]^T$) as $\mathbf{z}$ and the covariance matrix as Q, the population level probability is then calculated for each pair as

$$\log p(\boldsymbol{\nu}_p|\Delta) = -\frac{1}{2}(\boldsymbol{\nu}_p-\mathbf{z})^T Q^{-1}(\boldsymbol{\nu}_p-\mathbf{z}) - \frac{1}{2}\log|Q| - \frac{6}{2}\log 2\pi. \tag{30}$$

The gradient is also computed for each parameter in Δ. Similarly as for the likelihood computation, the partial derivative for parameters affecting the mean is

$$\frac{\partial}{\partial \Delta_j}\log p(\boldsymbol{\nu}_p|\Delta) = \frac{\partial \mathbf{z}^T}{\partial \Delta_j} Q^{-1}(\boldsymbol{\nu}_p-\mathbf{z}), \tag{31}$$

and for parameters that affect the covariance matrix Q the partial derivative with respect to parameter Δ_j is

$$\frac{\partial}{\partial \Delta_j}\log p(\boldsymbol{\nu}_p|\Delta) = -\frac{1}{2}(\boldsymbol{\nu}_p-\mathbf{z})^T Q^{-1}\frac{\partial Q}{\partial \Delta_j}Q^{-1}(\boldsymbol{\nu}_p-\mathbf{z}) - \frac{1}{2}\,\mathrm{Tr}(Q^{-1}\frac{\partial Q}{\partial \Delta_j}) \ . \tag{32}$$

For the set of all cell-specific parameters $\Theta = \{\boldsymbol{\nu}_p\}_{p=1}^{n_{\text{pairs}}}$ the total probability of the population level is then

$$\log(p(\Theta|\Delta)) = \sum_{p=1}^{n_{\text{pairs}}} \log p(\boldsymbol{\nu}_p|\Delta) \tag{33}$$

1.2.3 Prior and hyperprior distributions

The parameters τ_S and ρ_{SIS} are global parameters, and in the Bayesian framework we require prior distributions for these values. For the fluctuation timescale parameter τ_S we use a log-normal distribution with location parameter u_S and scale parameter s_S

$$p(\tau_S) \qquad = \frac{1}{\tau_S\sqrt{2\pi s_S^2}} \exp\left(-\frac{\log(\tau_S) - u_S}{2s_S^2}\right). \tag{34}$$

$$\tag{35}$$

We use $u_S = 1$, $s_S = 1$, which reflects previous studies that have found transcriptional bursting parameters operate on the order of one to several hours [3, 4]. The parameter τ_S (and its prior) is transformed into unbounded space using an exponential function. We assume a uniform prior on ρ_{SIS} between -1 and 1 (i.e. $\rho_{\text{SIS}} \sim \mathcal{U}(-1, 1)$). The parameter ρ_{SIS} (and its prior) is transformed into unbounded space using the sigmoid function $S(x) = 1/1 + e^{-x}$.

For the population level parameters $(m, \Lambda_S, \Lambda_R, s, \lambda_{\text{SIS}}, \Sigma_S, \Sigma_R)$ we also require prior distributions (hyperpriors). We assume uniform distributions for the mean and standard deviation parameters of the log-normal distributions. For the correlation parameter λ_{SIS} we use the LKJ distribution [5] with $\omega = 4$, which is defined as

$$\text{LkjCorr}(\lambda_{\text{SIS}}|\omega) \propto |\lambda|^{(\omega-1)}. \tag{36}$$

(When $\omega = 1$ the density is uniform, but when $\omega > 1$ there is an increasingly sharp peak around zero with increasing ω, and hence this expresses scepticism of high correlations between variables). The parameter λ_{SIS} is transformed into unbounded space with the tanh function $\tanh(x) = (1 - e^{-2x})/(1 + e^{-2x})$.

1.2.4 Inference with HMC

Collecting the terms for the three levels (bioluminescent time series, population level, prior/hyperprior), the total posterior of the parameters of the model given the bioluminescent time series is proportional to $\mathcal{L}(D|\theta)p(\Theta|\Delta)p(\tau_S)p(\rho_{\text{SIS}})$. To sample from this posterior distribution we constructed a Markov chain that has the posterior distribution as its stationary distribution. We use HMC for efficient parameter exploration, which uses Hamiltonian dynamics to produce distant proposals for the Metropolis algorithm and thereby explores parameter space more efficiently than a simple diffusive random walk [6].

To achieve efficient sampling the parameter space is augmented with fictitious momentum variables, and the dynamics of the system is conceptually equivalent to an object sliding on a frictionless surface with varying height. The state of the system is given by the position q and momentum p, and the potential energy $U(q)$ and the kinetic energy of the object $K(p)$ must be defined. The potential energy $U(q)$ of the object is defined to be minus the log probability of the posterior. The kinetic energy of the object $K(p)$ is defined as

$$K(p) = p^T M^{-1} p/2, \tag{37}$$

where M is a symmetric, positive-definite "mass matrix", and we use the identity matrix. The system is then full described by the Hamiltonian $H(q,p)$, and the evolution of both q and p are dictated by Hamilton's equations

$$\frac{dq_i}{dt} = +\frac{\partial H}{\partial p_i} \tag{38}$$

$$\frac{dp_i}{dt} = -\frac{\partial H}{\partial q_i} \tag{39}$$

At each step, the momentum variables are sampled from a Gaussian distribution (with covariance M) and then a trajectory is solved numerically using the leapfrog algorithm [6]. The leapfrog algorithm preserves volume in position-momentum phase space and hence reduces the error in the numerical trajectory. The final parameters at the end of the trajectory are then used for a Metropolis update, which typically has a high probability of acceptance but may be distant from the previous state.

We sampled the posterior with HMC sampling using the leapfrog integrator provided by the MATLAB 2017b function 'hmcSampler'. For each trajectory the step size is randomly sampled up to a maximum of 0.01 and number of steps is randomly sampled to a maximum of 100 steps.

1.2.5 Validation of the algorithm and performance of the inference

We applied our inference method on synthetic data in order to validate its ability to reliably estimate parameters. We simulated data sets with 3 different values for each of $\tau_S, \rho_{\mathrm{SIS}}, m, \Lambda_S, \Lambda_R, s, \lambda_{\mathrm{SIS}}, \Sigma_S$, and Σ_R.

The synthetic data consists of 50 pairs of cells with the same cell-cycle length as the *Dstn* gene data set, which represents a clone with medium cell cycle length (Supplementary Fig. 16). To generate the synthetic data, all parameters were fixed at their default values and one parameter was changed at a time. For the parameters $\tau_S, m, \Lambda_S, \Lambda_R, s, \Sigma_S, \Sigma_R$ the default parameter used represents the average across all genes measured experimentally (represented by the middle value within each panel of Supplementary Fig. 8 *i.e.* the blue circle in the middle of each panel), and for ρ_{SIS} and λ_{SIS} we used a default value of 0.4.

We simulated 50 pairs cells by first drawing cell-specific parameters from the population model (Equation 29). Once these cell-specific parameters had been generated, we simulate the time series by sampling multivariate normal distributions specified by Equations 12- 13. Overall the parameters were recovered with good accuracy, with the true value contained within the 90% credible intervals for all parameters.

1.2.6 Creating datasets with randomised pairings of cells

To test the contribution of the cell cycle to ρ_{SIS} we created a dataset consisting of randomised pairings. The cell-cycle lengths are more similar between sister cells than random pairings, and to control for this we wanted to choose cells to create a population with the same cell-cycle length correlation as sister cells. To achieve this, we first calculated the variance of differences in cell cycle lengths between sister cells. We then

sampled a normal random variable with this variance and then paired each cell with a non-sister cell with a difference in cell cycle length that was closest to the normal random variable.

1.2.7 Inferring parameters for non-sister cells in the same microenvironment

To investigate the origins of ρ_{SIS} we also created additional datasets with non-sister cells that, on average, had the same intercellular distance as the sister cells. As the cells are not necessarily born at the same time, the measurements of the earlier cell before the birth of the second cell were excluded. As an example, if cell 1 was born m time points before cell 2 (and the total number of data points in cell 1 is N_1) then the time series of cell 1 is $\mathbf{y}_1 = [y_m(t_m), y_{m+1}(t_{m+1}), ..., y_1(t_{N_1})]^T$ while the data points for cell 2 remain unaltered $\mathbf{y}_2 = [y_2(t_1), y_2(t_2), ..., y_2(t_{N_2})]^T$. The measurements of both cells from pair p are then combined into a single vector as before $\mathbf{y}_p = [y_m(t_m), ..., y_1(t_{N_1}), y_2(t_1), ..., y_2(t_{N_2})]^T$. The mean vector $\mathbf{m}_p$ is similarly adjusted *i.e.* $\mathbf{m}_p = [\langle R_1(t_m)\rangle, ..., \langle R_1(t_{N_1})\rangle, \langle R_2(t_1)\rangle, ..., \langle R_2(t_{N_2})\rangle]^T$.

1.2.8 Calculating the decay in correlation

For a given set of parameters it is possible to use the model to predict the decay in correlation between two sister cells over time. To select parameters we use the mean of the HMC posterior samples for each gene. Within the model there is a lower level describing single-cell dynamics and a higher level describing the distribution of cell-specific parameters across the population. There are accordingly two terms that represent the covariance between two cells, and there are two parameters that act to link two sisters: ρ_{SIS} and λ_{SIS}. The law of total covariance can be used to combine these two terms:

$$\text{cov}(R_1, R_2) \qquad = \text{E}(\text{cov}(R_1, R_2 \mid \mu_1, \mu_2)) + \text{cov}(\text{E}(R_1 \mid \mu_1), \text{E}(R_2 \mid \mu_2)), \tag{40}$$

The first term is from the stochastic single-cell dynamics and is evaluated from the model using the cross-covariance between two cells (Equation 13), which is evaluated from 0 to 13.5 hours (the average cell cycle length). The second term is from the correlation in cell-specific transcriptional activities between sisters. To calculate the correlation the covariance is then divided by the variance across all cells

$$\text{corr}(R_1, R_2) \qquad = \frac{\text{cov}(R_1, R_2)}{\text{Var}(R)} = \frac{\text{E}(\text{cov}(R_1, R_2 \mid \mu_1, \mu_2)) + \text{cov}(\text{E}(R_1 \mid \mu_1), \text{E}(R_2 \mid \mu_2))}{\text{E}[\text{Var}(R \mid \mu)] + \text{Var}(\text{E}[R \mid \mu])}. \tag{41}$$

In the model, we use the same correlation coefficient between the the sister cells at at time $t = 0$ (corr($R_1(0)$,$R_2(0) \mid \mu_1, \mu_2$), corr($R_1(0)$,$S_2(0) \mid \mu_1, \mu_2$), corr($S_1(0)$,$S_2(0) \mid \mu_1, \mu_2$)), which was chosen so that the total correlation between R_1 and R_2 from the model matched the empirical correlation coefficient between sister cells from the data at time $t = 0$.

2 Supplementary Tables and Figures

	Primer sequence (5' – 3'):
**F1**	CGA AGA GTA ACC GTT GCT AGG
**R1**	TGC TTC AAG TAG TGT GTG CC
**F2**	CGT GGC TGA ATG AGA CTG G
**R2**	CGT CTG TTG TGT GAC TCT GG
**Long Adaptor**	CGA AGA GTA ACC GTT GCT AGG AGA GAC CGT GGC TGA ATG AGA CTG GTG TCG ACA CTA GTG G
**Short Adaptor**	AAT TCC ACT AGT GTC GAC ACC AGT CTC TAA TTT TTT TTT TCA AAA AAA

Supplementary Table 1: Primers used for splinkerette PCR

	G1	S+G2+M	mitosis	Inferred S+G2	Total cell cycle
average [h]	3.29	9.63	0.70	8.93	12.92
% of cell cycle	25.47	74.53	5.44	69.09	100
CV	0.33	0.24	0.25		0.19

Supplementary Table 2: Cell cycle phase lengths. n=208 (FUCCI measurements); n= 41 (Mitosis duration measurements by luminescence microscopy).

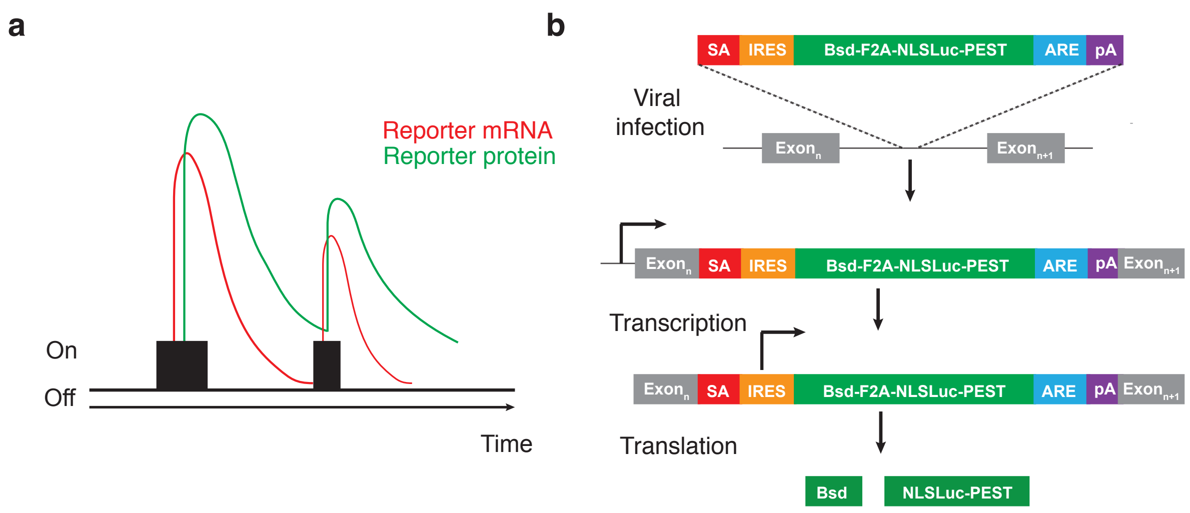

Supplementary Figure 1: a) A short-lived luciferase expressed from a short-lived mRNA closely mirrors fluctuations in transcriptional activity. b) Gene trapping strategy allowing to control NLSLuc-PEST expression by endogenous gene regulatory elements. SA- splice acceptor; IRES- internal ribosomal entry site; Bsd- blasticidin deaminase; F2A- co-translationally cleaved peptide; NLS- nuclear localisation signal; PEST-PEST sequence; ARE- AU-rich element; pA- poly A signal.

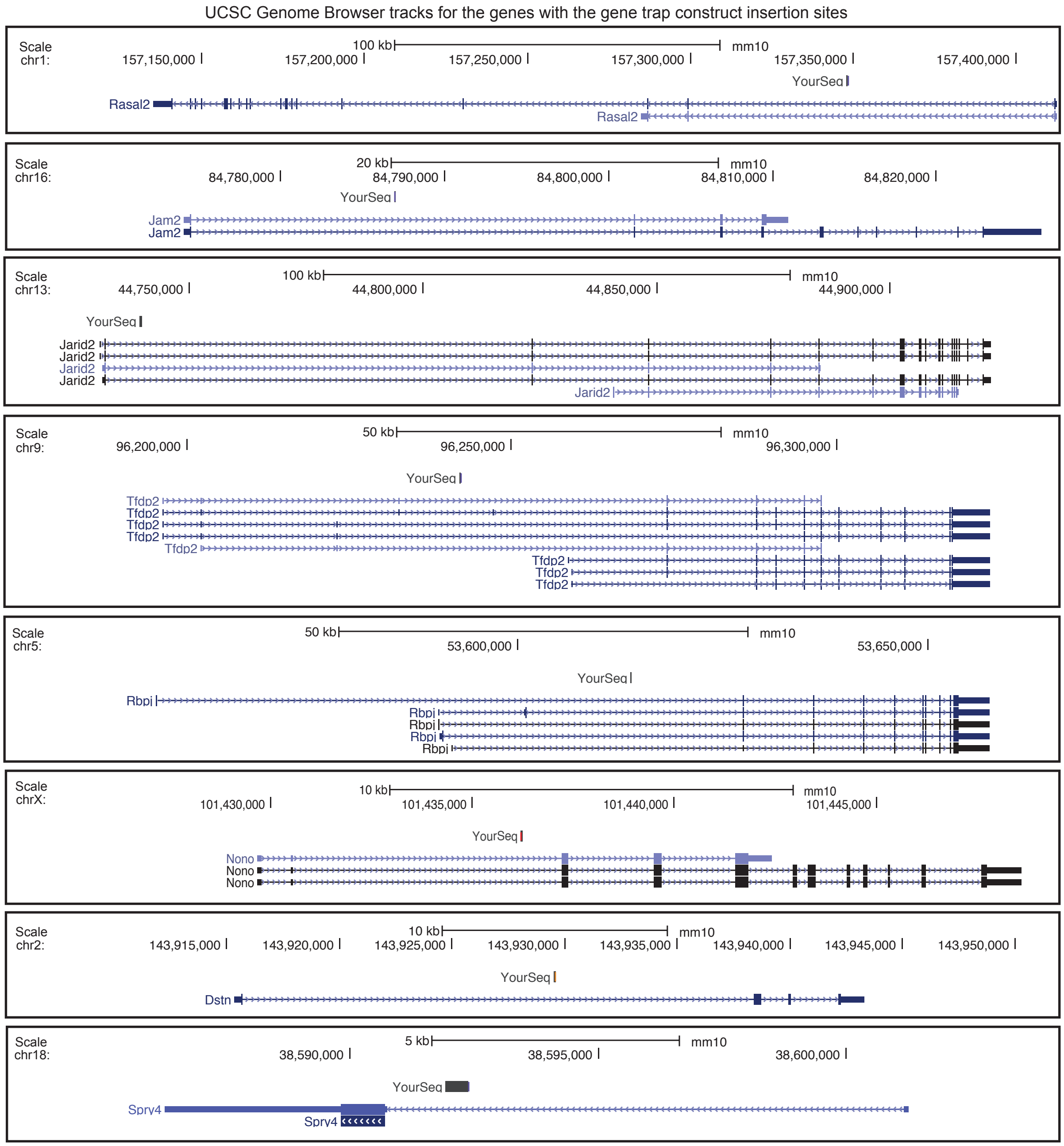

Supplementary Figure 2: UCSC Genome Browser tracks of endogenous genes with the gene-trap insertions. The insertion sites were mapped using splinekerette PCR and the exact genome locations are labelled with YourSeq.

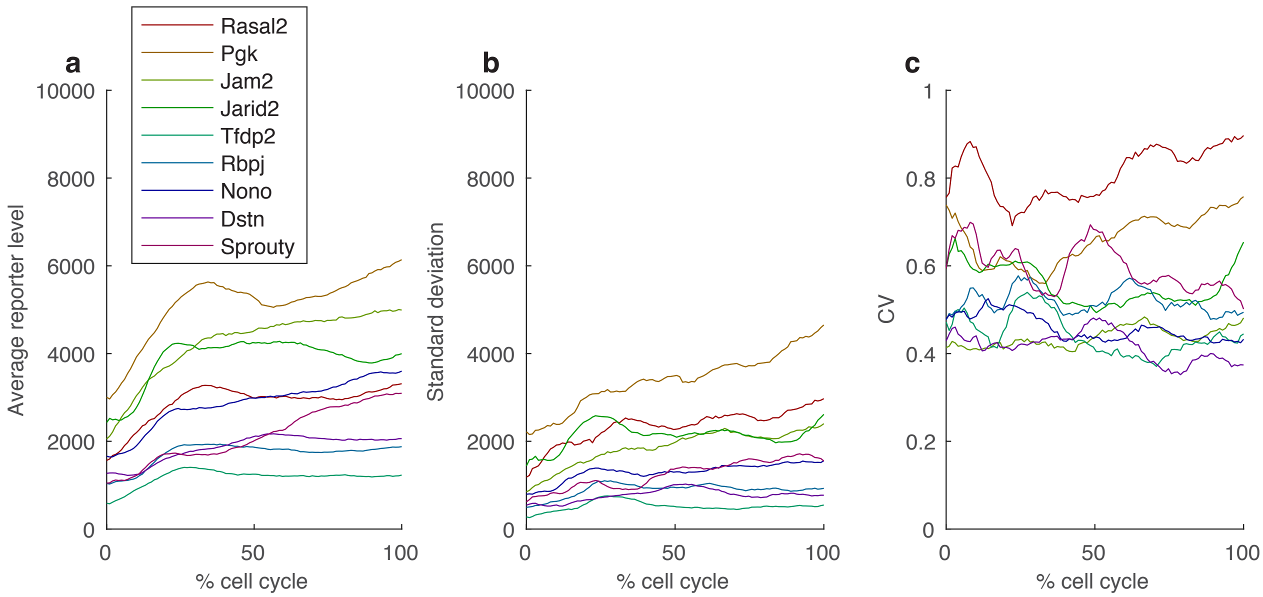

Supplementary Figure 3: a) Average reporter expression levels over the cell cycle for all genes (time is expressed in % of cell cycle time using spline interpolation). b) Standard deviation over the cell cycle for all genes. c) Coefficient of variation (CV) over the cell cycle for all genes.

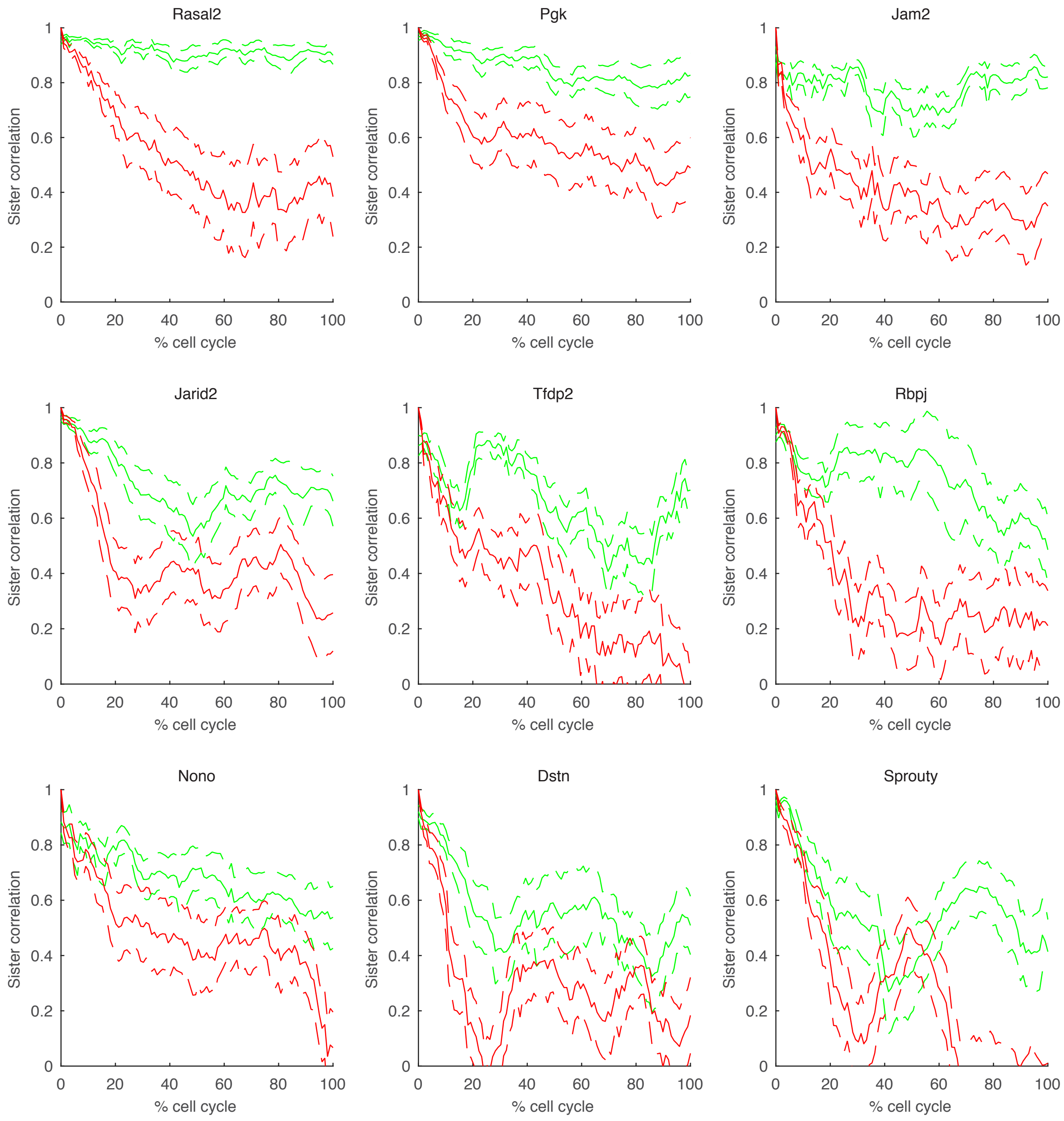

Supplementary Figure 4: The decrease in correlation between sister cells over the cell cycle for all genes measured (time is expressed in % of cell cycle time using spline interpolation). Green: correlation between sister cells; red: correlation between random cells, where each cell is matched with a non-sister with the nearest initial values. Error bars denote standard deviations obtained with bootstrap sampling.

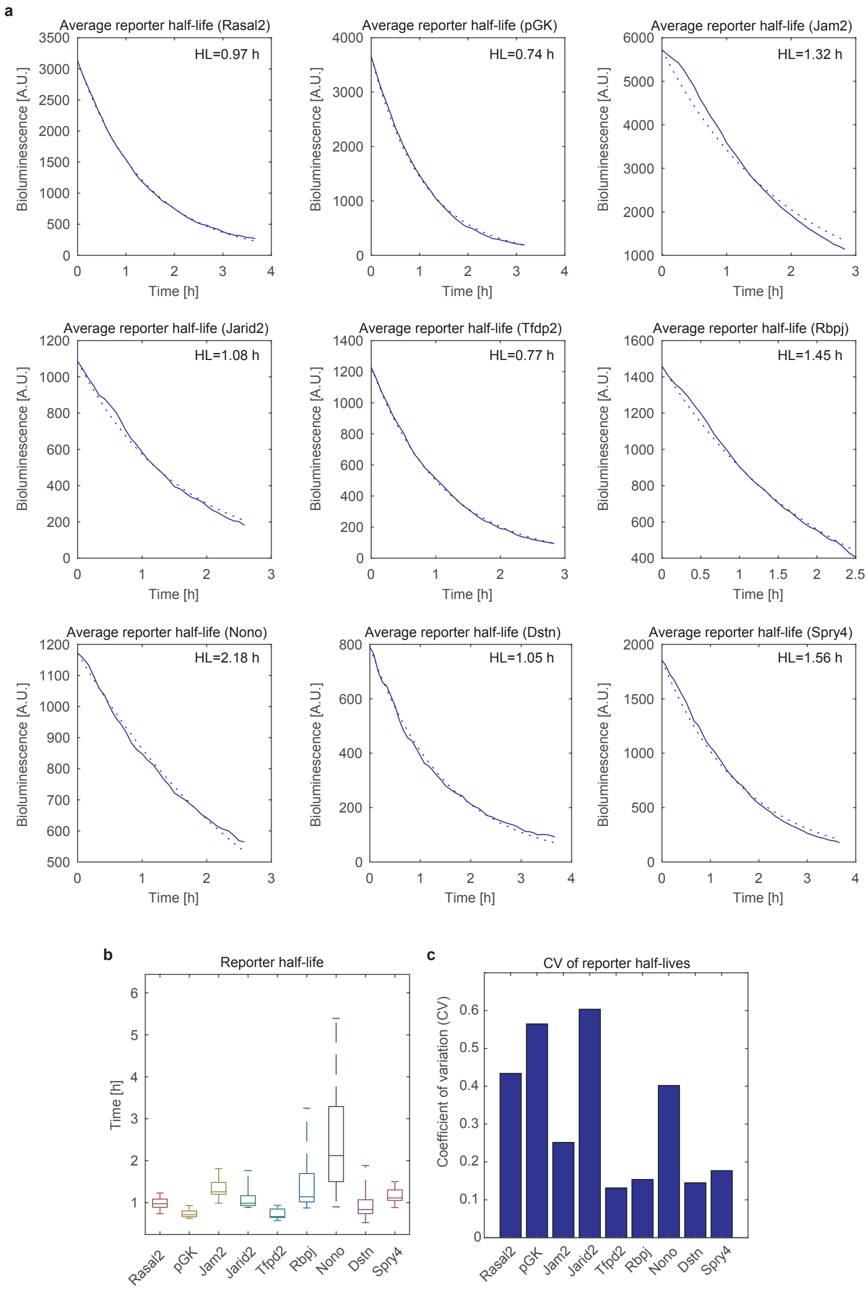

Supplementary Figure 5: Determination of the reporter half-lives. a) Reporter decay after actinomycin D treatment. Dashed blue line: first order exponential fit. HL: reporter half-life. (Rasa12: n=20 cells; pGK: n=25 cells; Jam2: n=44 cells; n=Jarid2: n=19 cells, Tfpd2: n=20 cells; Rbpj: n=50 cells; Nono: n=20 cells; Dstn: n=19 cells; Spry4: n=20 cells). b) Distribution of single cell reporter half-lives. Boxes: 25th, median (50th) and 75th percentiles of the posterior distributions; whiskers: 5th and 95th percentiles. c) coefficients of variation of single cell reporter half-lives. (Rasa12: n=20 cells; pGK: n=25 cells; Jam2: n=44 cells; Jarid2: n=19 cells, Tfpd2: n=20 cells; Rbpj: n=50 cells; Nono: n=20 cells; Dstn: n=19 cells; Spry4: n=20 cells).

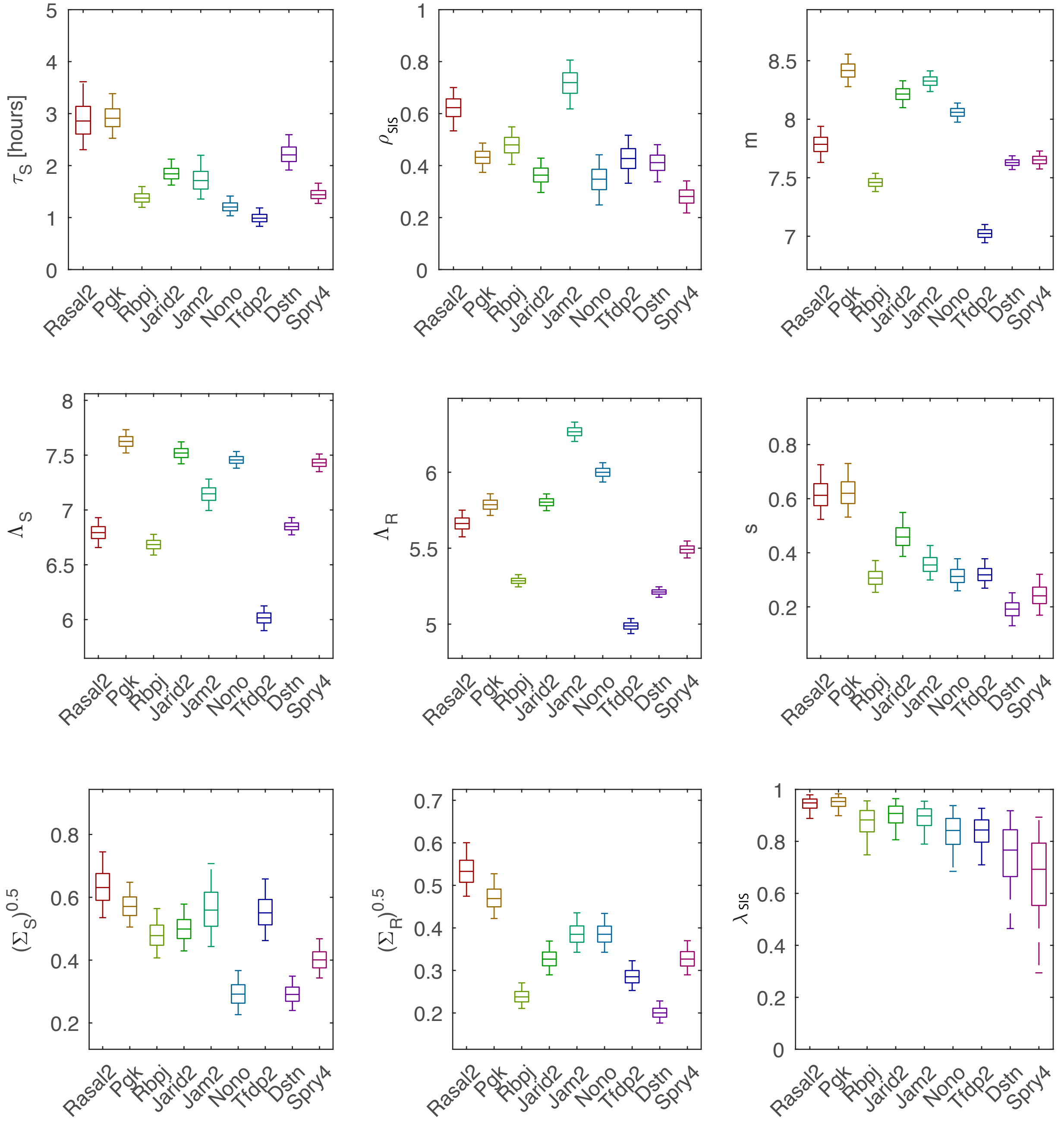

Supplementary Figure 6: Boxplots of parameter posterior distributions for all genes studied. The posterior distributions were estimated with 10,000 HMC samples for each gene. Boxes: 25th, median (50th) and 75th percentiles of the posterior distributions; whiskers: 5th and 95th percentiles.

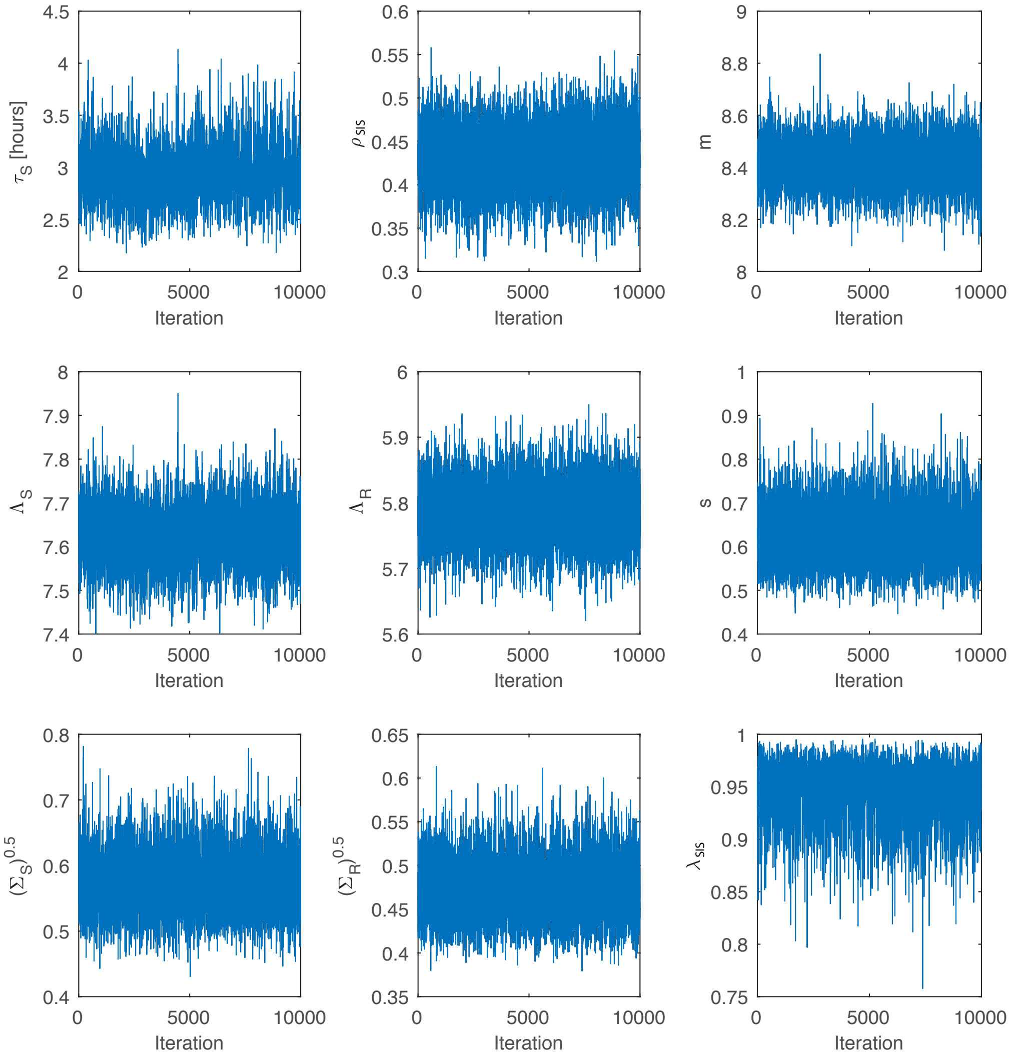

Supplementary Figure 7: Posterior chains of parameters for the pGK gene. The total chain is a combination of 4 chains with 2,500 iterations in each chain.

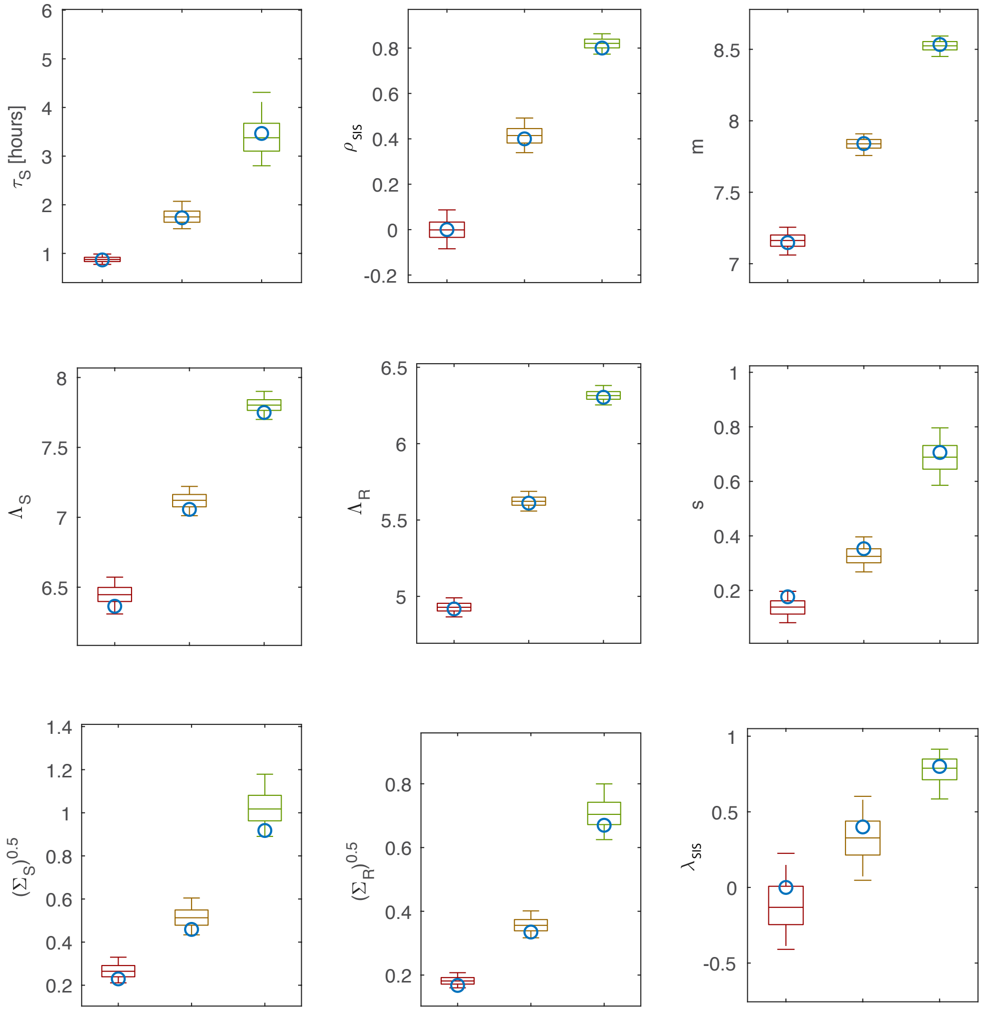

Supplementary Figure 8: Boxplots of parameter posterior distributions for synthetic data, where the true value is represented with a blue circle. The synthetic data consists of 50 pairs of cells with the same cell-cycle length as the *Dstn* gene data set, which represents a clone with medium cell cycle length. To generate the synthetic data, all parameters were fixed at their default values and one parameter was changed at a time. The default parameter set for all 9 parameters is represented by the middle value within each panel (*i.e.* the blue circle in the middle of each panel). The posterior distributions were estimated with 2,000 HMC samples for each synthetic data set. Boxes: 25th, median (50th) and 75th percentiles of the posterior distributions; whiskers: 5th and 95th percentiles.

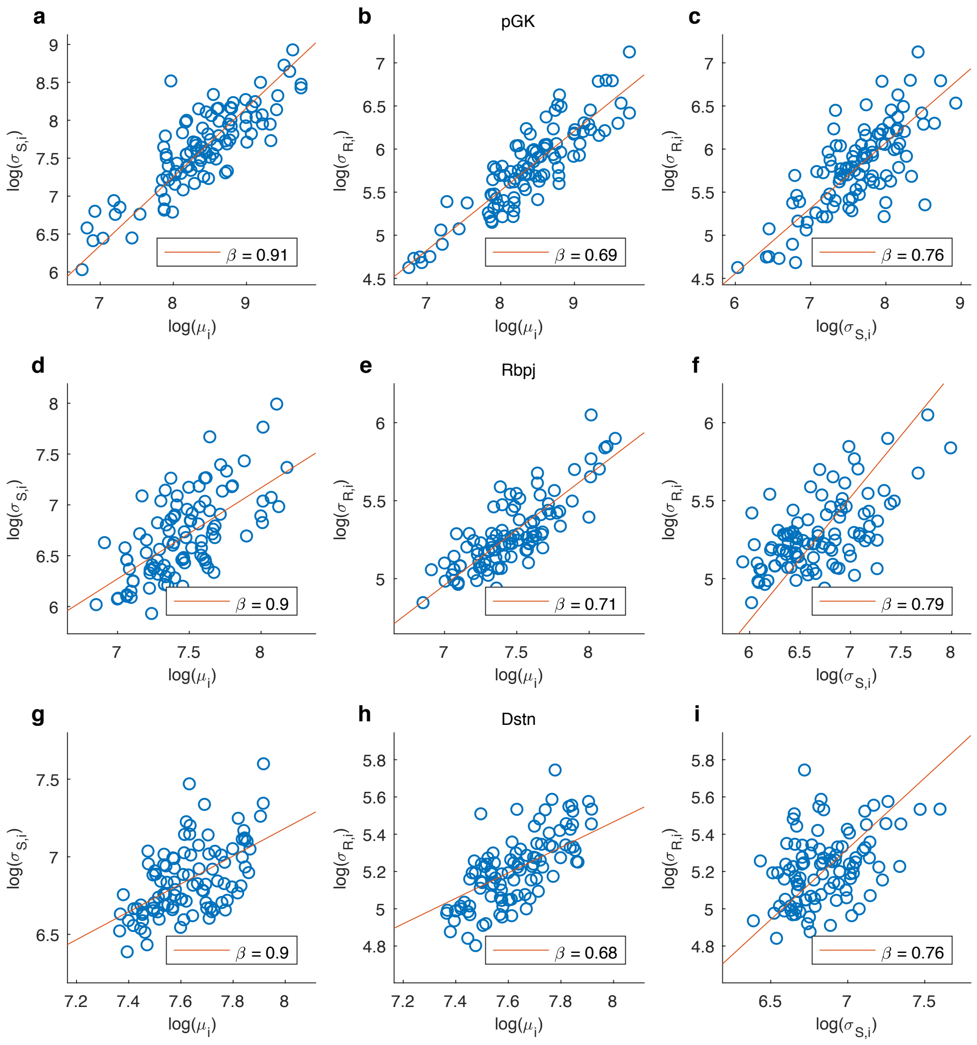

Supplementary Figure 9: (a,d,g) The posterior mean of μ_i and $\sigma_{S,i}$ for each individual cell for the genes pGK, Rbpj and Dstn. (b,e,h) The posterior mean of μ_i and $\sigma_{R,i}$ for each individual cell for the genes pGK, Rbpj and Dstn. (c,f,i) The posterior mean of $\sigma_{S,i}$ and $\sigma_{R,i}$ for each individual cell for the genes pGK, Rbpj and Dstn. The red line shows a linear regression with intercept zero and with slope β.

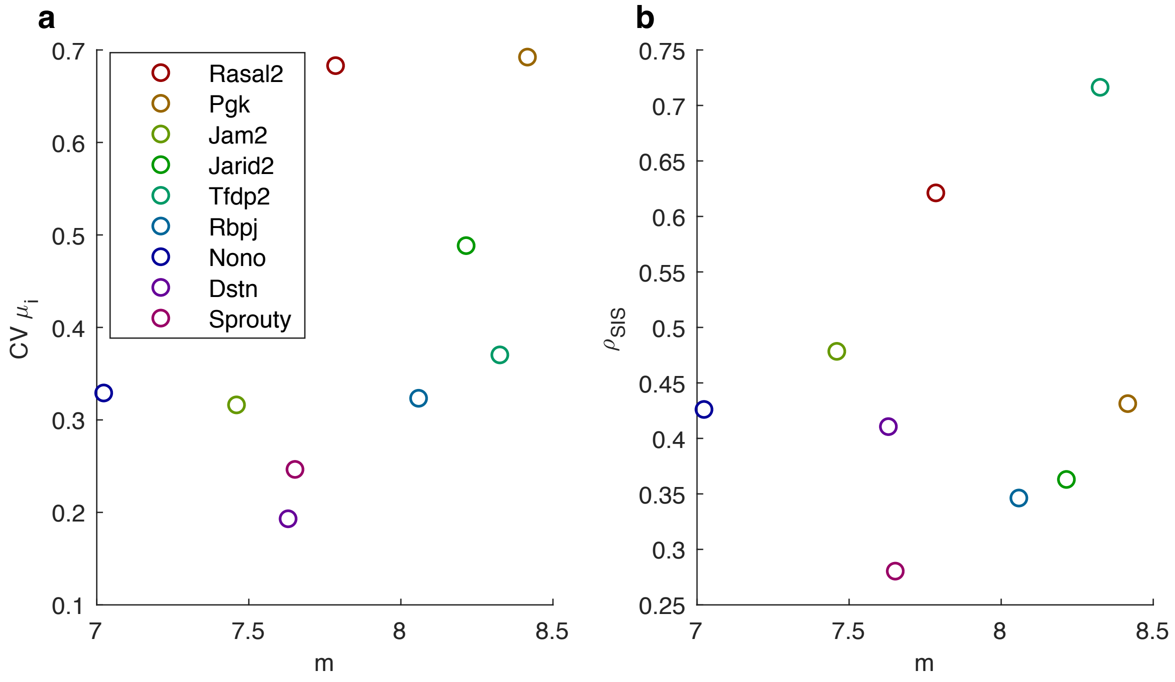

Supplementary Figure 10: a) Mean of the posterior distributions of the coefficient of variation (CV μ_i) for cell-specific means (calculated from the posterior distributions of s and m for each gene), against the mean m (in log space) of the expression level for each gene. b) Mean of the posterior distributions of the similarity in dynamics ρ_{SIS}, against the mean m (in log space) of the expression level for each gene.

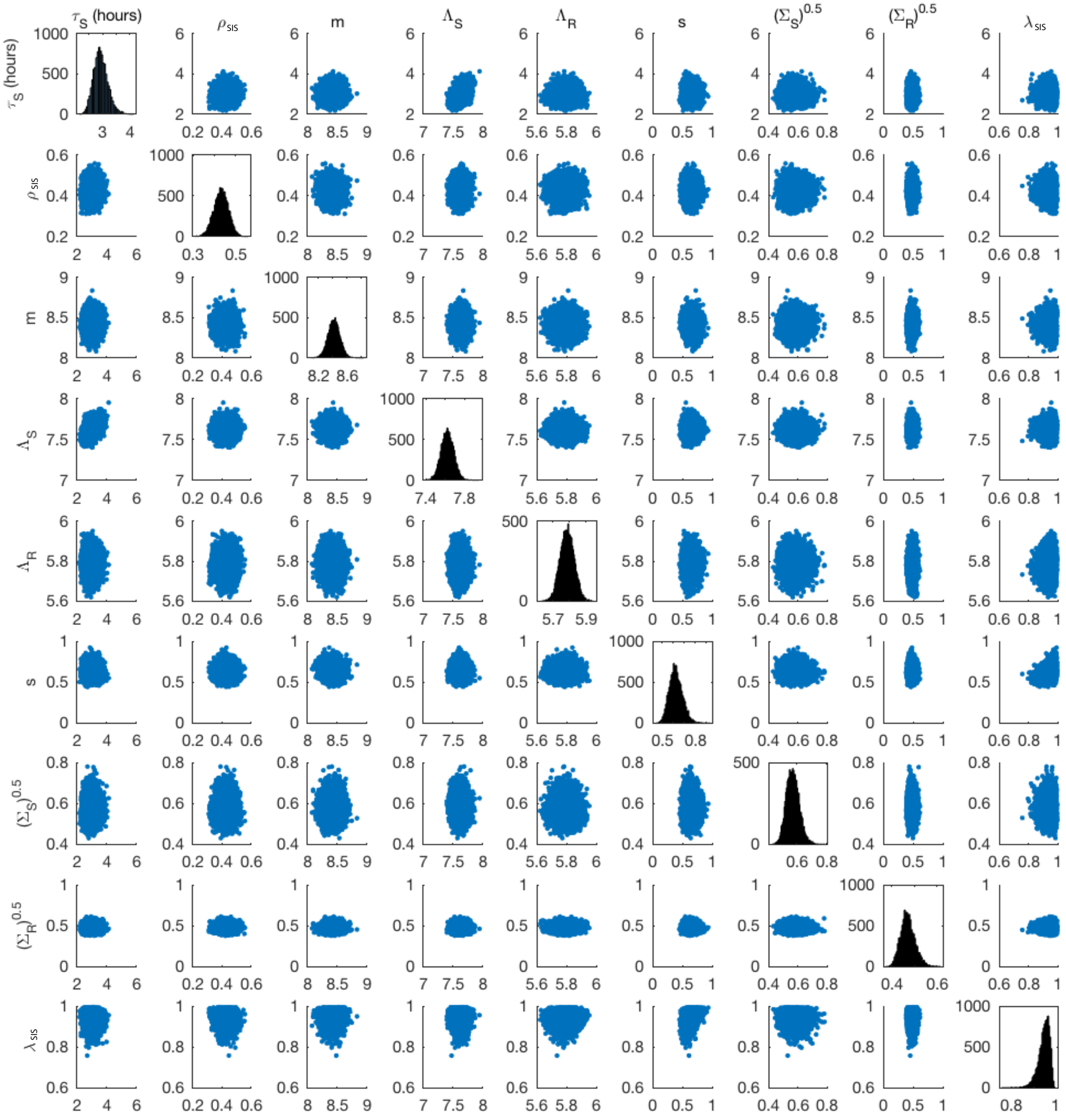

Supplementary Figure 11: All HMC parameter samples for the pGK gene. Diagonal plots represent histograms for a single parameter. Off-diagonal plots are scatter plots of two parameters at a time.

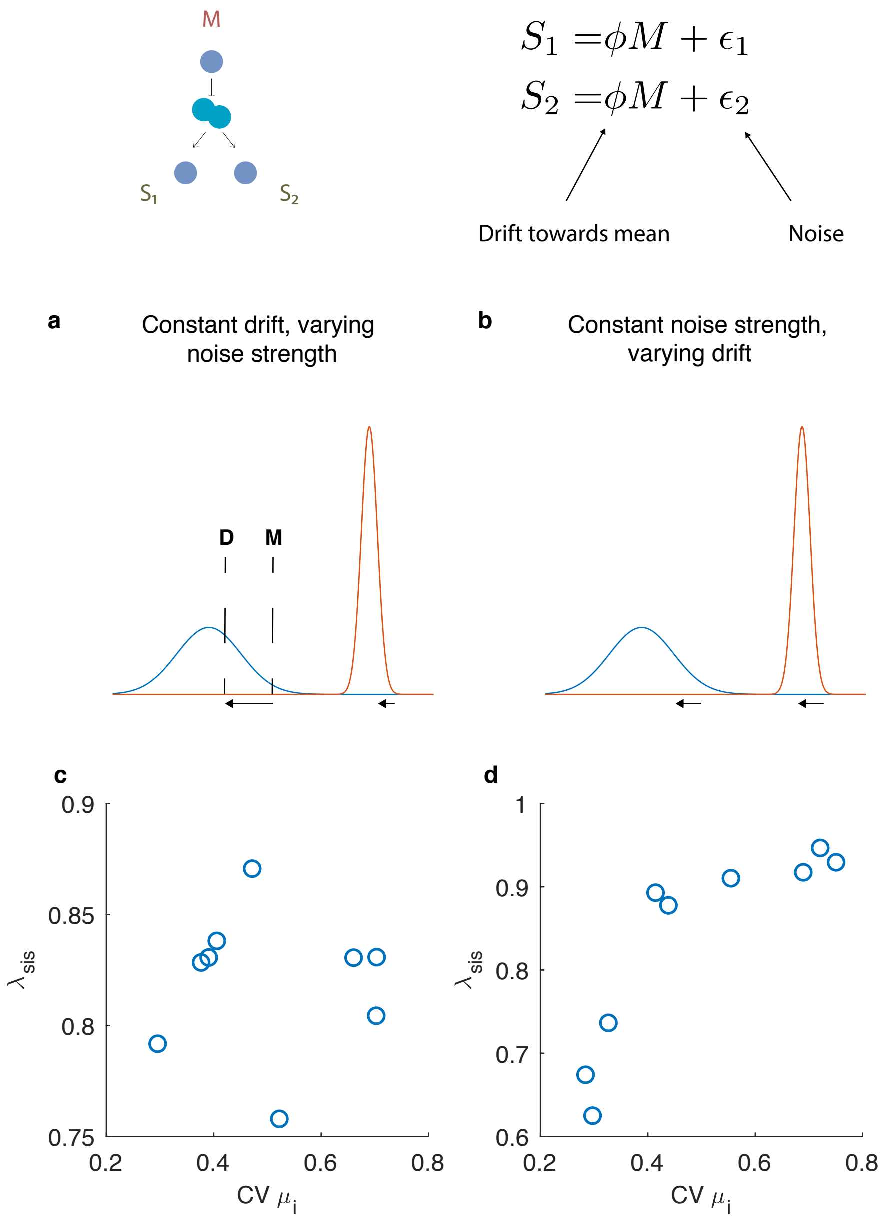

Supplementary Figure 12: Illustration of a simple model for the transmission of transcriptional activity across generations. The two daughter cells (S_1 and S_2) receive a fraction ϕ of the mother's transcriptional activity (M) plus Gaussian noise ϵ (in log space). Note that this is equivalent to an AR(1) process. (a) When ϕ is constant and the noise varies, there is no correlation between λ_{SIS} and $CV\mu_i$, as shown in (c). (b) When ϕ can vary but the noise is constant, there is positive correlation between λ_{SIS} and $CV\mu_i$, as shown in (d).

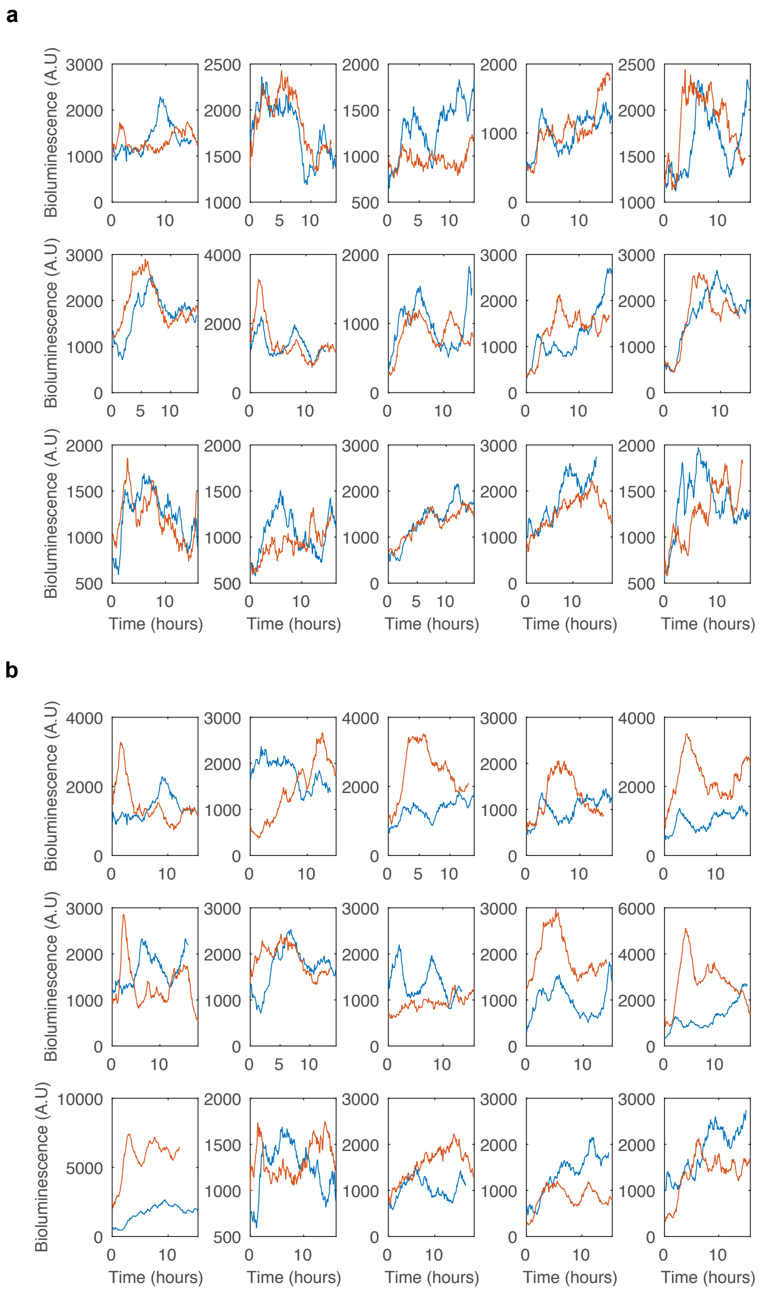

Supplementary Figure 13: a) Examples of 15 pairs of sister cells for the *Rbpj* gene. b) Examples of 15 pairs of cells for the *Rbpj* gene with randomised pairings of cells.

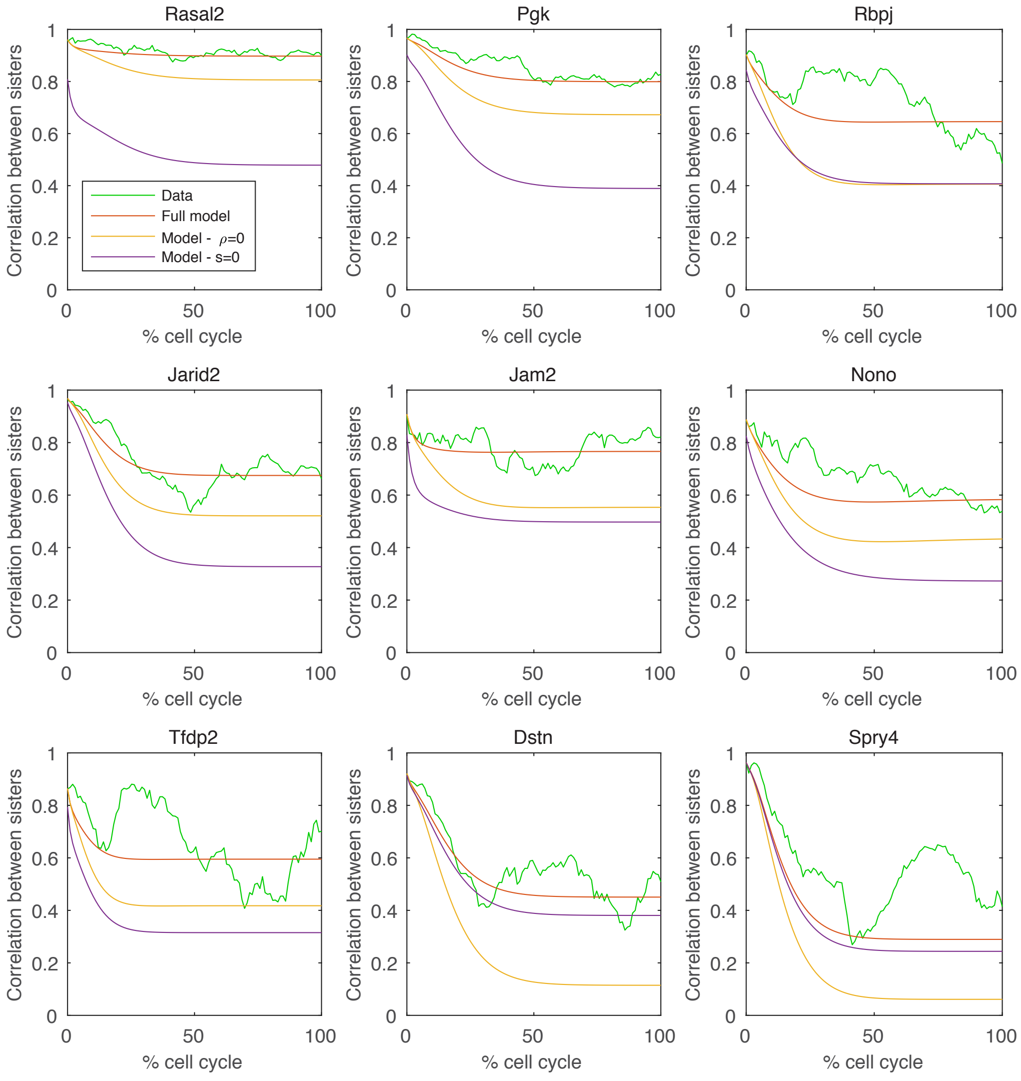

Supplementary Figure 14: Comparison of the decrease in correlation between sister cells over the cell cycle predicted by the model and the data. Green - the evolution of the sister cell correlation over the cell cycle from the data, where time is expressed in % of cell cycle time using spline interpolation. Red - the parameter posterior means for each gene are used to predict the evolution of sister-sister correlation over the cell cycle from the model, which is normalised to cell-cycle time by dividing by the average cell-cycle length (13.5 hours). Yellow - the correlation between sisters is recalculated from the model with $\rho_{\rm SIS} = 0$. Violet - the correlation between sisters is recalculated from the model with $s = 0$, which removes cell-specific means from the model.

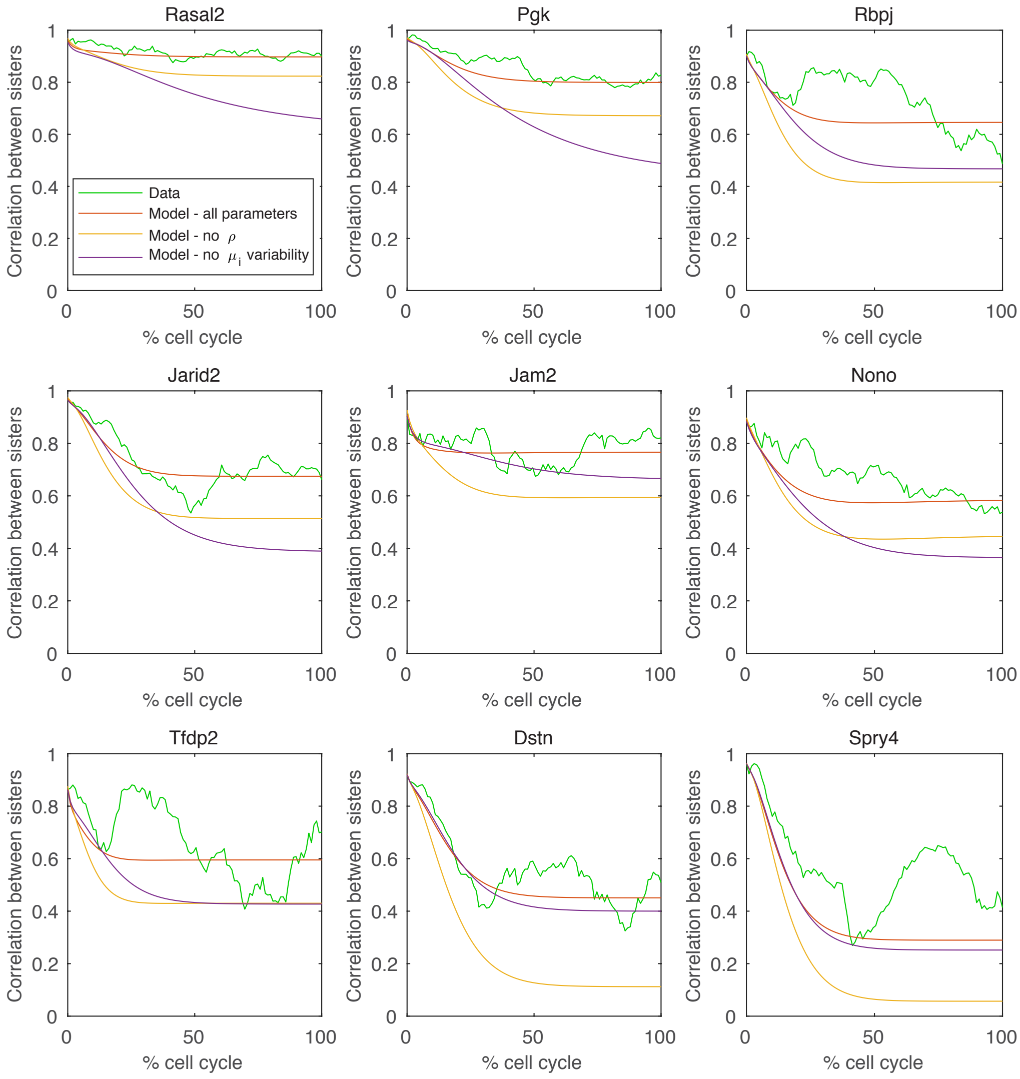

Supplementary Figure 15: Comparison of the decrease in correlation between sister cells over the cell cycle predicted by the model and the data. Green - the evolution of the sister cell correlation over the cell cycle from the data, where time is expressed in % of cell cycle time using spline interpolation. Red - the parameter posterior means for each gene are used to predict the evolution of sister-sister correlation over the cell cycle from the model, which is normalised to cell-cycle time by dividing by the average cell cycle length (13.5 hours). Yellow - the correlation between sisters from the model when the parameter ρ_{SIS} is removed from the model before fitting to the data. Violet - the correlation between sisters from the model when cell-mean variability is removed from the model (i.e. $s = 0$) before fitting to the data.

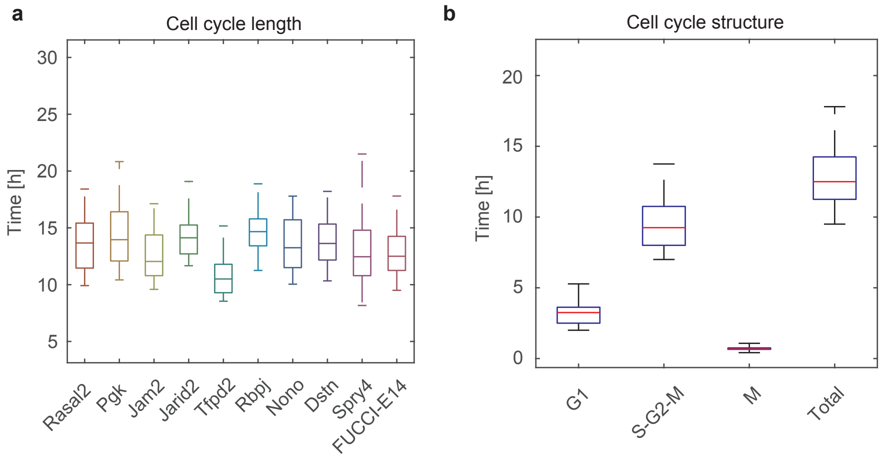

Supplementary Figure 16: Determination of cell-cycle phase lengths. a) Average cell cycle duration for the different cell lines; b) Average duration of G1, S-G2-M and M phase. Boxes: 25th, median (50th) and 75th percentiles of the posterior distributions; whiskers: 5th and 95th percentiles.

References

[1] C Gardiner. *Stochastic methods.* Springer Verlag, Berlin, 4th edition, 2009.

[2] C.E Rasmussen and C.K.I Williams. *Gaussian Processes for Machine Learning.* MIT Press, 2006.

[3] Benjamin Zoller, Damien Nicolas, Nacho Molina, and Felix Naef. Structure of silent transcription intervals and noise characteristics of mammalian genes. *Molecular systems biology*, 11(7):823, 2015.

[4] David M Suter, Nacho Molina, David Gatfield, Kim Schneider, Ueli Schibler, and Felix Naef. Mammalian Genes are Transcribed with Widely Different Bursting Kinetics. *Science*, 332(iii):472 – 474, 2011.

[5] Daniel Lewandowski, Dorota Kurowicka, and Harry Joe. Generating random correlation matrices based on vines and extended onion method. *Journal of Multivariate Analysis*, 100(9):1989–2001, 2009.

[6] Radford M Neal. MCMC using Hamiltonian dynamics. In S. Brooks, A. Gelman, G. L. Jones, and X.-L. Meng, editors, *Handbook of Markov Chain Monte Carlo.* CRC Press, New York., 2011.
